# Poor implementation of tobacco control measures and lack of education influences the intention to quit tobacco: a structural equation modelling approach

**DOI:** 10.1186/s12889-022-13565-3

**Published:** 2022-06-15

**Authors:** Mir Faeq Ali Quadri, Tenny John, Damanpreet Kaur, Maryam Nayeem, Mohammed Khaleel Ahmed, Ahmed M. Kamel, Santosh Kumar Tadakamadla, Vito Carlo Alberto Caponio, Lorenzo Lo Muzio

**Affiliations:** 1 Department of Preventive Dental Sciences, Dental Public Health, Jazan University, PO Box: 114, 45142 Jazan, Saudi Arabia; 2 Department of Maxillofacial Surgery and Diagnostic Sciences, Jazan University, 45142 Jazan, Saudi Arabia; 3 Department Oral Medicine and Radiology, BJS Dental College and Hospital, Ludhiana, India; 4 Department of Pharmacology, Jazan University, 45142 Jazan, Saudi Arabia; 5 Department of Oral Medicine and Radiology, M.N.R Dental College, Sangareddy, India; 6 Clinical Pharmacy and Pharmacy Practice Department, Cairo University, Cairo, Egypt; 7 School of Medicine and Dentistry & Menzies Health Institute Queensland, Griffith University, QLD Gold Coast, Australia; 8 Department of Clinical and Experimental Medicine, University of Foggia, 71122 Foggia, Italy

**Keywords:** Quit tobacco, Adults, Tobacco control measures, Developing nations, India, Structural equation model, Association

## Abstract

**Background:**

Tobacco consumption remains a public health issue and is one of the major causes of death in India. This study presents a validated conceptual model to assess the interaction between education, perceived application of tobacco control measures, type of tobacco and their effects on the intention to quit tobacco. Additionally, the direct and mediating roles of tobacco use -frequency, -duration, and -dependency on the intention to quit is also investigated.

**Methods:**

An analytical cross-sectional study was carried out, and data from tobacco users of six randomly selected states in India was collected via face-to-face interviews. Structural equation modeling (SEM) was performed using R v 3.6.3 to test the model fit and to explore the association between tobacco control measures and the intention to quit tobacco.

**Results:**

From 1962 tobacco users, 43.7% wanted to quit tobacco immediately. Tambakoo (57.7%) was the most common type of tobacco used and 68.9% said that minors could buy tobacco. Findings from SEM showed that that one standard deviation (SD) increase in the perceived application of tobacco control measures is directly associated with a 0.181 SD increase in the intention to quit tobacco (B = 0.181, *P* < 0.001), and this effect was partially mediated by frequency of tobacco consumption (B = 0.06, *P* < 0.05). Also, a better education level was associated with a higher intention to quit tobacco (B = 0.14, *P* < 0.001).

**Conclusions:**

To conclude, the application of tobacco control measures and a better education level may positively affect the intention to quit tobacco. The frequency of tobacco use and the number of influencers play an essential role in deciding to quit. In future, longitudinal studies are recommended to further substantiate the evidence.

**Supplementary Information:**

The online version contains supplementary material available at 10.1186/s12889-022-13565-3.

## Background

The World Health Organization (WHO) multiprong Monitor, Protect, Offer, Warn, Enforce, and Raise (MPOWER) project is a successful campaign aimed at protecting the population from the global tobacco pandemic [1, 2]. The MPOWER program is ratified by the WHO Framework Convention on Tobacco Control (FCTC) to combat the tobacco demand and supply in a nation. Some effective actions include; adopting price and tax measures to reduce the demand for tobacco (Article 6), regulating the packaging and labelling of tobacco products (Article 11), banning tobacco advertising, promotion and sponsorship (Article 13), offering people help to end their addictions to tobacco (Article 14), and banning sales to and by minors (Article 16). Implementation of these tobacco control policies have significantly lowered smoking prevalence and increased the quit ratio in 27 high-income countries [3]. However, tobacco consumption remains a public health issue. The 2020 Global Burden of Diseases study estimated that over 7 million deaths worldwide are associated with tobacco use [4]. Mortality rate among users is predicted to further increase to nearly 8 million per year by 2030. 80% of these deaths are expected from lower- and middle-income countries (LMIC’s) [5], as they account for nearly 80% of the world’s smokers [6].

India, a lower middle-income country (LMIC) with 1.35 billion people, has 274.9 million smokers, classified as the second-largest consumer of tobacco in the world [7]. Currently, over 267 million 15-year- and older adults use tobacco in India, 199 million consume smokeless tobacco (Tambakoo), 100 million smoke tobacco and 32 million use both [8]. Tobacco use is the major cause of death in India and over 1.135 million people have died due to diseases associated with tobacco consumption [9, 10]. Many nations signed the memorandum of understanding towards tobacco control, published evidence indicating the implementation of the tobacco control measures from lower- middle-income countries (LMICs) is limited [11, 12]. In the South-east Asian region (SEARO), India is one of the three countries to report a reduction in the prevalence of tobacco use; from 34.6% to 2009 to 28.6% in 2017, the other two being Bangladesh and DPR Korea [10]. Additionally, the recent national survey indicate a 6% decline in tobacco use among people of age 15-years- and older from 2016 to 2017 [8]. Nonetheless, further reduction in tobacco use is warranted, which may require strategic actions and timely implementation of strict regulations. Specific multiprong regulations that can change tobacco consumption pattern in India include raising taxes, designating smoke-free public places, controlling cigarette advertising, issuing direct health warnings and supporting prompt diagnosis and access to treatment of tobacco-related morbidities [13, 14]. These regulations have the capacity to influence tobacco quitting.

The intention to quit tobacco among tobacco users is a strong predictor of making an attempt towards actual quitting as highlighted by the Theory of Planned Behavior [15, 16], and there is currently no conceptual model that associates tobacco control measures with the Intention to Quit tobacco. This study aimed to report the perception of tobacco users on the implemented tobacco control measures and to investigate its influence on their Intention to Quit tobacco. A validated conceptual model is created to assess the interaction between education, perceived application of tobacco control measures, type of tobacco and their effects on the intention to quit tobacco is developed. The study also assesses the direct and mediating roles of tobacco use -frequency, -duration, and -dependency on the intention to quit. It was hypothesized that poor implementation of tobacco control measures and lack of basic education influences the frequency/duration of tobacco use, creating a high-level nicotine dependency, thereby preventing an individual from quitting tobacco use.

## Methods

### Study design and participant selection criteria

In this cross-sectional study, Indian nationals aged 18 years or older attending randomly selected dental schools, and who are current tobacco users (self-reported tobacco consumers) were recruited. Those with heart diseases and/or respiratory illnesses were excluded because of the assumption that these indivudals’ health conditions make them more inclined to quitting tobacco.

### Sample size calculation and sampling technique

The sample size was determined using a prevalence of 19.6% reporting an intention to quit [17], the relative precision of 5%, power of 80% and with the design effect of 2. After compensating for 10% dropouts, the target was to recruit a minimum sample of 542 participants from the randomly selected states (regions) of India.

A multi-stage cluster random sampling technique was carried out. At first, the 28 States in India were categorized into the north, south, east, west and central regions of the country. Next, one state from each of these regions was randomly selected using a lottery method namely: Punjab (north), Kerala (south), Bihar (east), Maharashtra (west), and Madhya Pradesh (central). An additional state of Telangana which was not drawn from the random selection process was also included. This was because one of the authors who trained the interns (description provided later) was affiliated with a teaching institute in this state. In the next step, one dental teaching hospital affiliated with the government was randomly selected from each of the aforementioned states using a lottery method. Finally, all individuals who approached the diagnostic clinics between September 2019 and December 2019, and who satisfied the inclusion criteria were invited to participate in the study.

### Study tool and variables

Data for this study was gathered through an interviewer-administered questionnaire (Additional file 1). A interviewer-administered rather than self-administered questionnaire was preferred to address a possible high rates of illiteracy in the population and also because the responses could be clarified. The questions collected details on the respondents’ age at last birthday, sex at birth, level of education (no formal schooling, primary level, intermediate level, high school, graduation and post-graduation), living condition (rural, semi-urban, and urban) and employment status (unemployed, self-employed and employee for others). Education was graded using a Likert scale item (1 through 6) since the effect of an increase in the level of education was assessed rather than comparing all levels of education to a reference group [18].

#### Information on tobacco

Next, the questionnaire collected the following information on tobacco use: type(s) of tobacco used (cigarette, bidis, shisha, tambakoo and beetel quid), duration of tobacco use (less than 5 years, 6–10 years, 11–20 years and more than 20 years), influencer(s) who suggested the individuals to quit tobacco (parents, relatives, friends and healthcare providers) which was recategorized into the number of influencers (0, 1, 2, 3, 4 or more). The dependent variable was the intention to quit (ITQ) tobacco use. Information on this was collected using one question, “Do you plan to quit tobacco?“ The response was on a five-point Likert scale (never, not yet decided, sometime in the future, in the next 6 months, and now) with a higher score indicating a more positive attitude [19].

#### Information on nicotine dependency

The questionnaire also collected details on the intensity of physical addiction to nicotine using the Fagerstrom Test for Nicotine Dependence [20]. Six questions were asked: how soon after you wake up do you use tobacco (within 5 min, 6 to 30 min, 31 to 60 min, and after 60 min), do you find it difficult to refrain from tobacco use in places where it is forbidden? (no / yes), which tobacco would you hate most to give up (the first one in the morning, any other), how many times per day do you use tobacco (10 or less, 11 to 20, 21 to 30, and 31 or more), do you use tobacco more frequently during the first hours after waking than during the rest of the day (no/yes), and do you use tobacco when you are so ill that you are in bed most of the day (no/yes). The cumulative score ranged from 0 to 10 and the higher total indicated more intense physical dependence on nicotine.

Respondents were also asked about the signs and symptoms they think they could suffer upon quitting tobacco (restless, insomnia/sleep problems, increased appetite/hungry, dizziness, difficulty concentrating, depressed/Sad, coughing, constipation, anxious/nervous, angry/irritable/frustrated). Each response option used a five-point Likert scale (1 = no, 2 = slightly, 3 = mildly, 4 = moderate, and 5 = severe).

#### Information on the implemented tobacco control measures

Each participant was asked about their exposure to the tobacco control policies, by acknowledging potential violations of the existing laws. The corresponding questions were itemized as: tobacco that you usually buy, is sold by a certified company (yes / no); in your area, tobacco can be bought by a minor age person (less than 18 years) (yes/no); is the tobacco that you usually buy, taxed? (yes/no); I see signs of “NO SMOKING” or “NO TOBACCO” quite often (yes/no); I have been offered free tobacco (smoking/smokeless) quit-service by other people (yes/no); I have offered free tobacco (smoking/smokeless) quit-service to other people (yes/no); the packaging of tobacco that you usually buy, displays harmful warnings of tobacco use (yes/no); television and movies that you usually watch, display about the warnings of tobacco use (yes/no); I feel that the place where I live, have strict regulations for the use of tobacco (yes/no), I have seen tobacco advertised on streets / on television / during movies (yes/no); and the tobacco tax has been raised recently (within 5 years from now) (yes/no). Each implemented policy was awarded one point and the variable was kept as continuous.

#### Face and content validity of the data collection tool

The questionnaire was tested for its face and content validity. The questionnaire was first developed in English. To maintain semantic equivalence, questionnaires were translated into two local languages (Hindi and Malayalam) from the original English version by two bilingual dental professionals from the states of Punjab and Kerala, who knew Hindi and Malayalam languages, respectively. Hindi could be read and/or understood from four (Maharashtra, Madhya Pradesh, Bihar, and Punjab) of the five selected states, except Kerala. Later, two interns (dental trainee) who served as data collectors from the states (region) of Punjab and Kerala conducted the reverse translation from the local languages to English. Discrepancies were addressed by reaching a mutual consensus. In addition, 10 respondents in each state were administered the questionnaire to determine the ease with which they could respond to the questions, identify any ambiguity in any of the questions, acceptance of terminologies used, ease of administering the questionnaire, and subsequently, the questionnaire was tested for its reliability and validity.

To test the reliability of the tool, internal consistency and test-retest reliability were sorted. Cronbach α coefficient was computed for internal consistency, and a value of 0.70 and above was considered as internally consistent [21]. Test-retest reliability to assess the stability across time and the intra-class correlation coefficient (ICC) was computed and values were scored according to the prescribed criteria: <0.40-poor to fair, 0.41-0.60-moderate, 0.61-0.80-good, > 0.80-excellent [22]. Additionally, Kappa statistic was computed to check the extent of agreement between two consecutive administrations, and agreements followed the categorization: <0.20-poor, 0.21–0.40-fair, 0.41–0.60-moderate, 0.61–0.80-substantial, 0.81–1.00-almost perfect [23]. For the validity of the tool, discriminant and construct validity tests were computed. Discriminant validity was assessed by testing the tool across different educational levels using Kruskal-Wallis. For construct validity, the model fit was assessed and is described below.

#### Model structure and model fit

**Fig. 1:**
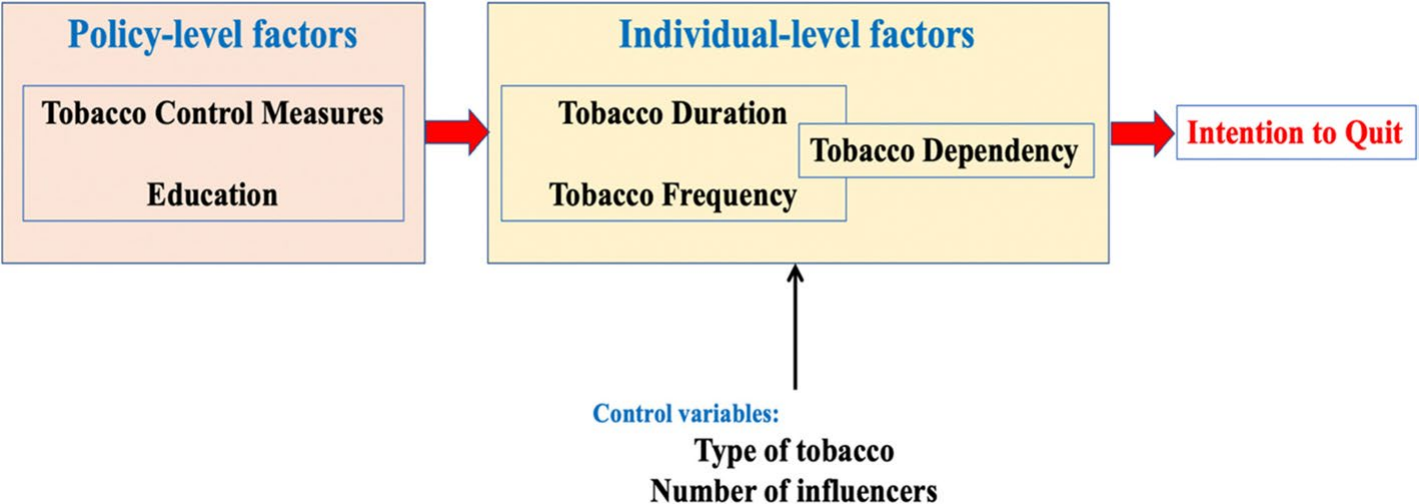
Proposed hypothetical model for intention to quit tobacco

The conceptual hypothesized model was tested using structural equation modeling (SEM). The model (Fig. 1) included two exogenous (Tobacco control measure score and level of education) and four endogenous (tobacco-dependency, -duration, and –frequency, and intention to quit) variables. It was hypothesised that effect of Tobacco control measures’ score and level of education on the intention to quit would be mediated by tobacco-dependency, -duration, and –frequency. The control variables included in the model comprise, number of influencers (people advising to quit tobacco), the category of influencer (none, healthcare professional, friends, or parents), sex (dichotomous variable), age and type of tobacco used (Fig. 1). The type of tobacco was dummy coded into five yes/no variables to take into account that some respondents used more than one type of tobacco. The five yes/no variables were included as covariates in the model. A similar approach was used for the category of influencers. The duration and frequency (overall) of tobacco use were also included as covariates. A full model was initially tested and non-statistically significant pathways were removed in a stepwise fashion to reduce the complexity of the model and improve model fit. The duration and frequency of tobacco use were allowed to freely co-vary due to the correlation between both variables (r = 0.33). The duration and frequency were included as continuous variables in the model with four levels in each variable and lower scores representing lower frequency or duration of tobacco use. Model fit was assessed using the following measures: Comparative Fit Index (CFI), Tucker Lewis Index (TLI), Chi-square statistics (C_min_), Root Mean Square Error of Approximation (RMSEA), RMSEA upper 90% CI, and Standard Root Mean Square Residual (SRMR). The cutoff values suggested by Hu and Bentler were used in assessing the model fit [24].

### Training of data collectors

Five dental interns, one each from their respective states, were trained to carry out the interviews. The trainees performed three mock interviews and in each, the trainer pretended to be the interviewee.

### Statistical analysis

Statistical analysis was performed using R v 3.6.3. Counts and percentages were used to summarize the categorical variables and mean ± standard deviation was used for continuous variables. Structural equation modelling (SEM) was used to validate the research model. Although initially included, sex was eliminated from the model as it was not associated with dependency, duration and frequency of tobacco use and the intention to quit. Total, direct and indirect effects were estimated, and the corresponding standard errors and corresponding 95% confidence intervals were estimated using 2000 bootstrapped samples [25]. Non-significant pathways were eliminated in a stepwise manner to obtain a better model fit. However, the mediation analysis pathways were not removed to test the indirect effects of frequency, dependency, and duration of tobacco use. Maximum likelihood was used in reporting the estimates of model parameters. Variables were checked for multicollinearity before their inclusion in the model. Ordinary least squares (OLS) linear regression was used to assess the association between sociodemographic characteristics and ITQ. Model fit was assessed using the fitted vs. residuals plot and normal Q-Q plot. The ITQ was included as a five-level continuous variable. Linear mixed modelling was used to assess the robustness of the results after including the state as a random intercept. The results from linear mixed modelling were compared to the results from OLS. Fisher-Exact test was used to assess the association between state and ITQ while one-way ANOVA with post-hoc pairwise comparisons was used to assess the association between states and tobacco control measures. Hypothesis testing was performed at a 5% level of significance. Analyses for structural equation modeling were performed using the lavaan package in R v 3.6.3, and plots were constructed using the lavaan Plot package. For this, a pathway framework is hypothesized and put to the test (Fig. 1).

## Results

### Findings from psychometric analyses

The Hindi and the Malayalam versions of the tool showed good internal consistency with Cronbach’s alpha estimates of 0.89 and 0.91, respectively. Their ICCs were greater than 0.90. A significant difference in response was observed across different education levels of the study participants (*p* < 0.001).

### Descriptive findings of the study

A total of 2294 respondents completed the questionnaires, though, only the 1962 completed questionnaires reporting the use of tobacco products were utilized for this study (response rate = 85.5%). The mean age of the included respondents was 43.2 ± 13.4 years. The socio-demographic characteristics of the respondents are shown in Table 1. About 1844 (94%) were males, 447 (22.8%) did not receive formal schooling, and 933 (47.5%) were self-employed and unemployed. Tambakoo (57.7%) was the most common type of tobacco used; 857 (43.7%) respondents wanted to quit tobacco use immediately. Participants were advised to quit tobacco use majorly by relatives (22.9%) and doctors (22.4%). A majority (47.1%) of the participants used tobacco five times or less per day.

**Table 1:** Descriptive statistics for the study sample (*N* = 1962)

Variables	N (%)
**Age**	43.2 (13.4%)
**State (Region)**	
Bihar	304 (15.5%)
Kerala	249 (12.7%)
Madhya Pradesh	252 (12.8%)
Maharashtra	589 (30.0%)
Punjab	459 (23.4%)
Telangana	109 (5.5%)
**Location**:	
Rural	613 (31.2%)
Semi-Urban	424 (21.6%)
Urban	925 (47.1%)
**Sex**:	
Males	1844 (94.0%)
Females	118 (6.01%)
**Education**:	
No formal schooling	447 (22.8%)
The primary level of schooling	544 (27.7%)
Intermediate level of schooling	500 (25.5%)
High school	245 (12.5%)
Graduation	210 (10.7%)
Post-graduation	16 (0.82%)
**Employment**:	
Unemployed	285 (14.5%)
Self –employed	648 (33.0%)
Employee for others	1029 (52.4%)
**Duration of Habit**:	
Less than 5 years	435 (22.2%)
6–10 years	641 (32.7%)
11–20 years	438 (22.3%)
More than 20 years	441 (22.5%)
**Person who asked to quit**:	
No one	474 (32.3%)
Parents	242 (16.5%)
Relatives	336 (22.9%)
Friends	86 (5.87%)
Health providers (Doctors/Dentists)	328 (22.4%)
**How soon after waking would you first use tobacco**:	
Within 31–60 min	1186 (60.4%)
Within 5–30 min	492 (25.1%)
Within 5 min	284 (14.5%)
**Do you find it difficult to refrain from tobacco use in places where it is forbidden?**	
No	1410 (71.9%)
Yes	548 (27.9%)
**Time of the day you prefer using tobacco**:	
Early morning	359 (18.3%)
During the day	772 (39.3%)
After every meal	744 (37.9%)
Nighttime before sleep	79 (4.03%)
**Daily frequency of smoking**:	
5 or less	922 (47.1%)
6–10 times	749 (38.2%)
11–20 times	253 (12.9%)
More than 20 times	35 (1.79%)
**Duration of smoking**:	
Less than 5 years	435 (22.2%)
6–10 years	641 (32.7%)
11–20 years	438 (22.3%)
More than 20 years	441 (22.5%)
**Daily frequency of Smokeless tobacco (n = 817)**:	
5 or less	491 (60.1%)
6–10 times	239 (29.3%)
11–20 times	79 (9.67%)
More than 20 times	8 (0.98%)
**Duration of Smokeless tobacco (n = 817)**:	
Less than 5 years	239 (29.26%)
6–10 years	256 (31.3%)
11–20 years	163 (20%)
More than 20 years	159 (19.5%)
**Have you used tobacco even when you were sick?**	
No	983 (50.1%)
Yes	975 (49.7%)
**Influencers to stop smoking**:	
No influencers	474 (24.2%)
One influencer	996 (50.8%)
Two influencers	225 (11.5%)
Three influencers	226 (11.5%)
Four or more influencers	41 (2.09%)
**Tobacco product used**	
Cigarette	674 (34.4%)
Bidis (or Equivalent – Rolled cigarettes)	715 (36.4%)
Shisha	44 (2.24%)
Smokeless tobacco (Tambakoo)	1132 (57.7%)
Betel quid	187 (9.53%)
Other products used	1.40 (0.73)
**Intention to quit**:	
Now	857 (43.7%)
In the next six months	375 (19.1%)
Sometime in the future, beyond six months	172 (8.77%)
Not decided	438 (22.3%)
Never	120 (6.12%)

Data was sumamrized as counts and percentages for categorical variables and mean ± SD for continuous variables

The variable was dummy coded as yes/no to take into account mixed users

### Findings from the perceived-application of tobacco control measures

Table 2 demonstrates the proportion of participants responding ‘yes’ to the application/observation of tobacco control measures in their daily life. About 1485 (75.7%) of respondents reported that tobacco was sold by a certified company, 1458 (74.5%) noted their tobacco was taxed, and more than three-quarter (78.3%) of the study population noted that the tobacco tax was high. Minors could buy tobacco in their respective states (regions) as stated by 68.9% respondents. More than a two-third (68.6%) of the respondents have been offered free tobacco and 1322 (67.4%) had offered free tobacco to others. Also, a majority of the participants (83.4%) had seen “No smoking” signs and warnings on packages and also tobacco advertisements, and 781 (39.8%) reported the States (region) had strict regulations on tobacco use.

**Table 2:** Application of perceived tobacco control measures

Tobacco Control Measures	N (%)
Tobacco sold by a certified company	1485 (75.7%)
Tobacco can be bought by a minor	1352 (68.9%)
Is your tobacco taxed	1458 (74.5%)
‘No Smoking’ signs	1816 (92.6%)
I have offered free tobacco quit-service	1345 (68.6%)
I have been offered free tobacco quit-service	1322 (67.4%)
Warning on package	1824 (93.0%)
Warning on TV	1837 (93.6%)
Have strict regulations	781 (39.8%)
Seen tobacco advertised	1637 (83.4%)
Tax raised	1536 (78.3%)

### Findings from tobacco dependency

**Fig. 2:**
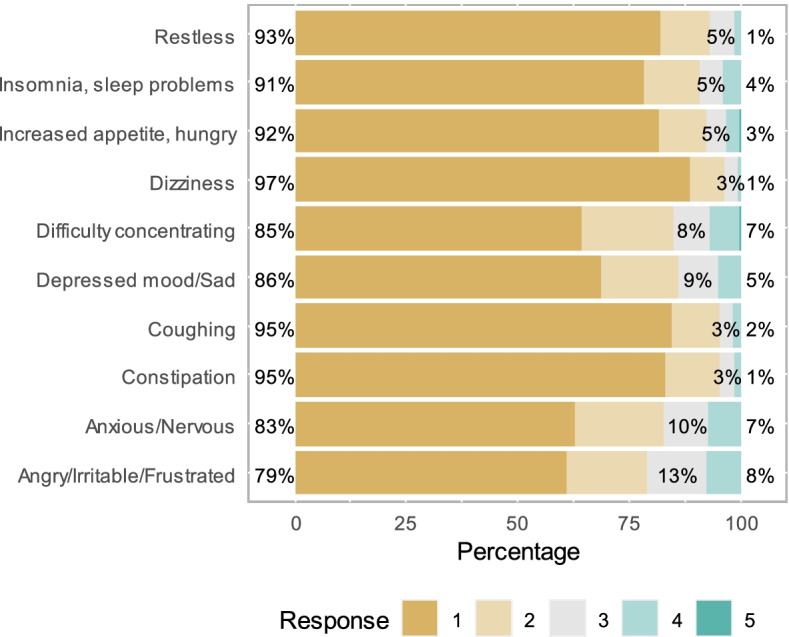
Distribution of dependency symptoms (1 = No symptoms, and 5 = Severe)

The majority of respondents anticipated none to mild tobacco dependency symptoms for each of the categories of symptoms explored - anxious/nervous, depressed/sad, difficulty concentrating, increased appetite, hungry, insomnia, restless, constipation, coughing, and dizziness (Fig. 2). The most severe symptom reported by 8% of respondents was being angry/irritable/frustrated.

Percentages on the left represent respondents with no and slight symptoms, and that on the right represent respondents with moderate and severe symptoms. Numbers in the middle represent respondents who reported mild symptoms.

### Findings from the structural equation modelling (SEM)

**Fig. 3:**
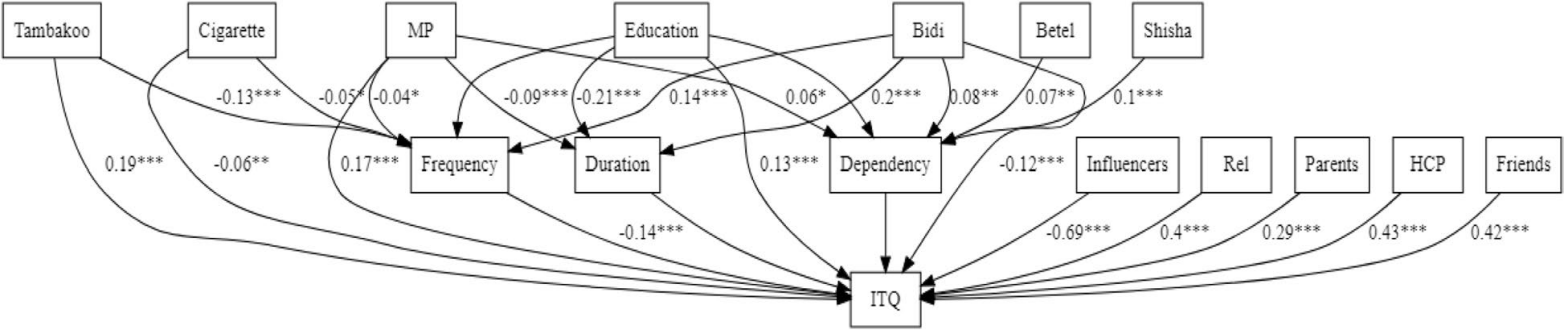
Pathway estimates for the structural equation model. MP: Tobacco control measures, ITQ: Intention to Quit; Rel: Relatives, Influencers: Number of influencers. Only significant pathways are shown (* *P* < 0.05, ** *P* < 0.01, *** *P* < 0.001)

The CFI and TLI were 0.967 and 0.922, respectively. The RMSEA and upper 95% confidence interval for RMSEA were 0.03 and 0.04, respectively. The SRMR was 0.016. These fit measures indicate that the data was a good fit for the hypothesized model. The SEM showed that the self-reported exposure to tobacco control measures was positively associated with the intention to quit (total effect = 0.188, *P* < 0.001). The direct effect of tobacco control measures accounted for the majority of its total effect (B = 0.181, *P* < 0.001), indicating that the application of tobacco control measures directly affects the intention to quit. The effect was also partially mediated by frequency of tobacco consumption (B = 0.06, *P* < 0.05). Neither duration nor dependency mediated the effect of tobacco control measures on the intention to quit (Fig. 3).

Education positively influenced the intention to quit tobacco (B = 0.14, *P* < 0.001). The direct effect of education accounts for > 90% of its effect (B = 0.13, *P* < 0.001) implying that better education was associated with a higher intention to quit. This effect was partly mediated by frequency of tobacco consumption (B = 0.006, *P* < 0.05).

Higher frequency of tobacco use (B = -0.257, *P* < 0.001) and use of bidis (B = -0.115, *P* < 0.05) and cigarettes (B = -0.058, *P* < 0.05) were associated with lower intention to quit tobacco. The duration of tobacco use (B = -0.028, *P* > 0.05) and degree of dependency (B = -0.035, *P* > 0.05) were not significantly associated with the intention to quit. Increase in the number of sources that provide advice regarding tobacco cessation (B = 0.686, *P* < 0.001) and use of tambakoo (smokeless tobacco) was associated with a higher intention to quit (B = 0.541, *P* < 0.001) when compared to any other tobacco.

The use of bidis (B = 0.142, *P* < 0.001) was associated with higher frequency of consumption while the use of cigarettes (B = -0.048, *P* < 0.05) and tambakoo (B = -0.135, *P* < 0.001) were associated with lower frequency of consumption (Table 3). The use of bidis was associated with longer duration (B = 0.199, *P* < 0.001) and higher dependency (B = 0.078, *P* < 0.01) when compared to other products. The use of betel quid (B = 0.07, *P* < 0.01) and shisha (B = 0.103, *P* < 0.01) were associated with higher dependency compared to the use of other products (Table 3). Results after using education as a multinomial variable are shown in Table S5 (Additional file 2).

**Table 3:** Effects of predictor variables on intention to quit tobacco, represented by the total- direct- and indirect-standardized coefficients

Pathway (IV ➔ DV)	Total effect	Direct effect	Indirect effects (Mediation)
Tobacco Control Measures ➔ Quit	0.176 ***	0.167***	006* (Frequency)
			0.002 NS (Duration)
			0.001 NS (Dependency)
Education ➔ Quit	0.14***	0.13***	0.006* (Frequency)
			0.005 NS (Duration)
			0 NS (Dependency)
Frequency ➔ Quit	-0.257***	-0.257***	Only direct effects are available
Duration ➔ Quit	-0.028 NS	-0.028 NS	
Dependency ➔ Quit	-0.035 NS	0.035 NS	
N Influencers ➔ Quit	0.686***	0.686***	Only direct effects are available
HCP vs. None ➔ Quit	0.425***	0.425***	Only direct effects are available
Rel vs. None ➔ Quit	0.399***	0.399***	
Friends vs. None ➔ Quit	0.417***	0.417***	
Parents vs. None ➔ Quit	0.288***	0.288***	
Bidis ➔ Quit	-0.115***	Only total effects were of interest as these variables were included as controlling factors	
Cig ➔ Quit	-0.058**		
Tambakoo ➔ Quit	0.186***		
Bidis ➔ Frequency	0.142***	0.142***	Only direct effects are available
Cig ➔ Frequency	-0.048*	-0.048*	
Tambakoo ➔ Frequency	-0.135***	-0.135***	
Bidis ➔ Duration	0.199***	0.199***	Only direct effects are available
Bidis ➔ Dependency	0.078**	0.078**	Only direct effects are available
Betel ➔ Dependency	0.07**	0.07**	
Shisha ➔ Dependency	0.103**	0.103**	

Results represent the standardized coefficients

* *P* < 0.05, ** *P* < 0.01, *** *P* < 0.001, *NS *Non-significant (*P* > 0.05)

N Influencers: Number of influencers

**Fig. 4:**
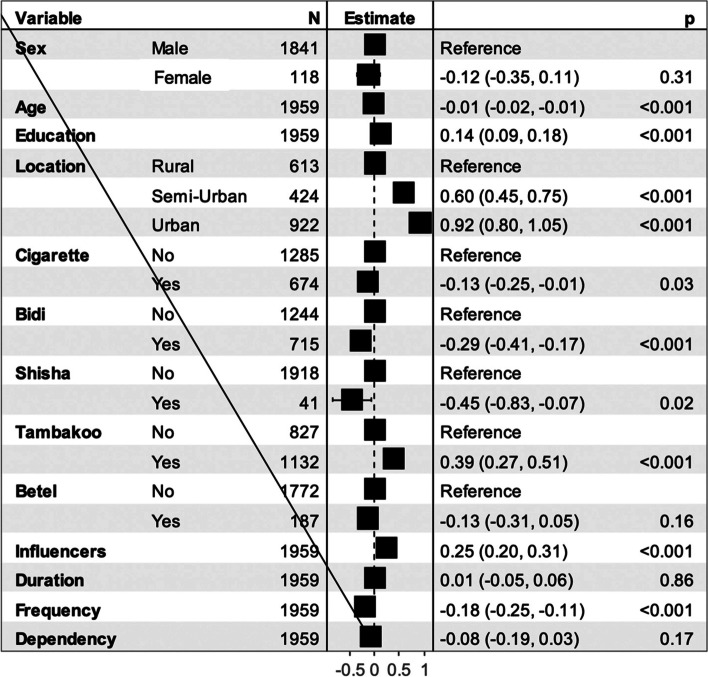
Forest-plot for the factors associated with the intention to quit tobacco

Linear regression analysis results (Fig. 4) showed that the intention to quit was higher in respondents from semi-urban (B = 0.59, *P* < 0.001) and urban areas (B = 0.93, *P* < 0.001) compared to respondents from rural areas. A year increase in age (B = -0.01, *P* < 0.001), higher number of reported influencers (B = 0.25, *P* < 0.001) and use of tambakoo (B = 0.39, *P* < 0.001) were also associated with ITQ. The use of shisha (B = -0.45, *P* < 0.05), cigarettes (B = -0.13, *P* < 0.05), and bidis (B = -0.29, *P* < 0.001), and greater frequency of tobacco use (B = -0.18, *P* < 0.001) were associated with a lower intention to quit. The use of betel quid and the duration and dependency score were not significantly associated with the intention to quit.

The analysis was performed using linear regression. Estimates (B) represent the average change in intention to quit.

A statistically significant association was observed between state and ITQ (*P* = 0.001). The proportion of respondents with the highest intention to quit were from Kerela, Bihar, and Punjab (Additional file 2: Fig. S2). One-way ANOVA showed that the average tobacco control measure score was significantly differenet between states (*P* < 0.001, Fig. S3). Post-hoc pair wise comaprisons (Additional file 2: Table S2) showed that the average tobacco control measures were significantly higher in Bihar and Kerela than Madhya prades and Maharashtra. Regarding the association between the type of influencer and ITQ (Additional file 2: Table S3), A statistically significantly association was observed between the influencer category and the ITQ (*P* < 0.001) with respondents who had no influencer showing the highest hesitancy. A statistically significant association between all ten dependency symptoms and the intention to quit was also observed (Additional file 2: Table S4). Among patients who wanted to quit now, the highest average scores were observed for angriness/irritability, anxiousness/nervousness, depressed mood/sadness, and difficulty concentrating.

## Discussion

According to the Global Adult Tobacco Survey (GATS), the influence of tobacco control measures is essential to upgrade the capacity and scale up the implementation of select demand reduction provisions of WHO FCTC [26]. In accordance with this, the current study validates a conceptual model and investigates the effects of the tobacco control measures on the intention to quit tobacco. Further, the influence of education level and, the mediating role of type, frequency of tobacco use and dependency is also assessed. It was observed that the intention to quit tobacco was strongly associated with the implemented tobacco control measures, and this relationship was partially mediated by the frequency of tobacco consumption. It implies, that the tobacco control measures if implemented effectively, may substantially increase the quitting rates. This is in agreement with the findings from most countries, where the lower prevalence of cigarette smoking was attributed to the effective implementation of tobacco control measures measures [27]. Nearly, 43.7% of the responders in this study stated that they wanted to quit tobacco immediately, and about 19% reported that they would quit within the next six months [12]. This estimate is somewhat similar to the findings from other LMICs such as Kenya (65%), Zambia (69%), Mexico (55%), and Mauritius (54%) and, also from high-income countries like Germany (60%), United Kingdom (62%) and France (65%) [28].

The current study identifies education as an essential factor. Better education level positively influences the intention to quit tobacco, and the direct effect of education is estimated to be more than 90%. This finding is consistent with at least two reports which indicate that the individuals educated till secondary level or higher demonstrated greater intention to quit tobacco [29, 30]. The association between education and tobacco use is further supported by data from national GATS surveys in India [7, 12] and studies from Malaysia and Poland [31, 32].

The responders from rural areas demonstrated a lower intention to quit in comparison to urban and semi-urban areas, and this could be attributed to the lower level of education and tobacco literacy in rural regions of India. Furthermore, evidence suggests that tobacco users living in rural areas have lower financial and psychological support, and this deprives them of making healthy life choices, including quitting tobacco [33, 34]. A wealth of studies suggest unequal distribution of income, education and healthcare services in India [35–40]. Whilst India is moving towards Universal Health Coverage [39], it is important to understand that the tobacco users with lower education level are less likely to avail themselves to essential healthcare oppurtunities [41, 42]. So, addressing the socioeconomic disparities and escalating the effective implementation of tobacco control measures can reduce the burden on health care facilities occurring because of tobacco-related illnesses [40].

Among the types of tobacco consumed, the bidi users demonstrated high dependency and lower intention to quit, and this is consistent with a nationwide study carried out in Bangladesh [43]. Also, evidence supports the claim that high nicotine dependency among bidi users in contrast to other tobacco products could be an important predictor of a weaker intention to quit [15, 44–47]. Bidi use in comparison to other tobacco products is a greater public health concern in India, as its sales in many rural districts of India are still observed to be informal, untraceable, and exempted from taxes [48].

In discussing the strengths, the current study presents a validated model to report the effects of tobacco control measures on intention to quit tobacco. The information is gathered directly from the tobacco users to present a ground-level observation of the implemented policies, and the findings substantiate the pieces of evidence which utilized information on the legislative implementation of tobacco control measures [49, 50]. Additionally, it is the first study to evaluate the role played by the educational level, in the relationship between the tobacco control measures and the Intention to Quit tobacco.

There are several limitations to this study, and the findings should be interpreted carefully. The current study uses a questionnaire-based assessment to identify the implementation of tobacco control measures which are not the actual MPOWER scoring scheme, however, the findings compliment it. The perception of responders may not provide precise assessment of the implemented tobacco control policies, and may not cover the true objective of the FCTC regulations. For instance, “I see signs of “NO SMOKING” or “NO TOBACCO” quite often” may not measure if people using tobacco where forbidden from its use. Because of its cross-sectional design, no cause and effect relationship between various factors and the intention to quit tobacco could be assumed. Next, the results may not be attributed to the entire population of tobacco users in India as only the individuals attending the dental clinics were approached. Nonetheless, the recruitment of participants involved a multistage-random sampling procedure. Because the study relied on perceived responses, the data collected may be subjected to recall bias and social desirability. Also, the study had significantly more male responders, and it is plausible that future studies with equal sex representation may display distinct findings. Lastly, the outcome variable did not collect the data on the actual abstinence from tobacco. However, evidence shows a strong association between intention to quit tobacco and its actual abstinence [15, 16, 51, 52].

Despite the limitations, the overall model and its findings serve as evidence for the public health specialists in LMICs to further promote, advocate and implement the tobacco control measures/policies to combat the tobacco epidemic. Also, the disparity in socioeconomic characteristics and its influence on tobacco consumption is a matter of grave concern and could be a reason for the rapid shift of tobacco consumption from high-income countries to the LMICs [30].

## Conclusions

This study observed a positive association between the perceived implementation of tobacco control measures, education level and intention to quit among tobacco users suggesting that the application of tobacco control measures along with better education positively affects the intention to quit. Also, the frequency of tobacco use and the number of influencers play an essential role in tobacco users decision to quit. The bidi users had the least intention to quit than the users of other tobacco types. In the future, longitudinal studies are recommended to further substantiate the effect of tobacco control measures on the intention to quit tobacco.

## Supplementary Information

**Additional file 1.** English version of the questionnaire.

**Additional file 2.** Sumplementary tables and figures.

## Data Availability

The data will be made available upon reasonable request made to the corresponding author.

## Abbreviations

CFI: Comparative Fit Index
HIC: High-income countries
ICC: Intra-class correlation
ITQ: Intention to quit (tobacco)
LMIC: Lower-and Middle-income countries
MPOWER: Monitor, Protect, Offer, Warn, Enforce, and Raise
SEM: Structural Equation Modelling
TLI: Tucker Lewis Index
RMSEA: Root Mean Square Error of Approximation
