# Supplementary material for: Poor implementation of tobacco control measures and lack of education influences the intention to quit tobacco: a structural equation modelling approach
Source: BMC Public Health. 2022 Jun 15;22:1199. doi: 10.1186/s12889-022-13565-3 (PMC9202196; doi:10.1186/s12889-022-13565-3)
Supplement: Supplementary file 1 — Additional file 1. English version of the questionnaire. [file 12889_2022_13565_MOESM1_ESM.pdf]

QUESTIONNAIRE – TOBACCO USE

1. Age: _______ (in years)

2. Location:
   A. Name of the Country ________________
   B. State/Territory ___________________
   C. Type
      a) Rural (1)
      b) Semi-urban (2)
      c) Urban (3)

3. Gender:
   A. Male (1)
   B. Female (2)

4. Level of education completed
   A. No formal schooling (0)
   B. Primary level of schooling (1)
   C. Intermediate level of schooling (2)
   D. High school (3)
   E. Graduation (4)
   F. Post-graduation (5)

5. Employment
   A. Unemployed (0)
   B. Self-employed (1)
   C. Employee for others (2)

6. What type of Tobacco do you use?
   A. Cigarette
   B. Beedis (or Equivalent – Rolled cigarettes)
   C. Shisha (pipe smoking)
   D. Tambakoo (Smokeless - Dipping)

DR. QUADRI - COPYWRITE

E. Betel nut (In pan)
F. COMBINATION:
____________+________________+_________________

7. Since how many years have you been using any form of tobacco?
   A. Less than 5 years (1)
   B. 6-10 years (2)
   C. 11-20 years (3)
   D. More than 20 years (4)

8. Who usually ask you to quit tobacco?
   A. No one (0)
   B. Parents (1)
   C. Relatives (2)
   D. Friends (3)
   E. Health providers (Doctors/Dentists) (4)

9. When do you plan to quit tobacco use?
   A. Now (1)
   B. In the next six months (2)
   C. Sometime in the future beyond six months (3)
   D. Not decided (4)
   E. Never (5)

DR. QUADRI - COPYWRITE

1. How soon after walking would you first use tobacco
   A. Within 5 minutes (3)
   B. Within 5-30 minutes (2)
   C. Within 31-60 minutes (1)

2. Do you find difficult to refrain from tobacco use in places where it is forbidden? (like religious-places, public-places, relatives or friends place etc.)
   A. Yes (1)
   B. No (0)

3. Which time of the day do you prefer using the tobacco the most?
   A. Early Morning (1)
   B. During the day (2)
   C. After every meal (3)
   D. Nigh time before sleep (4)

4. How many times do you use tobacco in a day
   A. 5 or less (0)
   B. 6- 10 times (1)
   C. 11 – 20 times (2)
   D. More than 20 times (3)

5. Have you used tobacco even when you were sick?
   A. Yes (1)
   B. No (0)

What happens if you quit tobacco OR do not use it for a long duration? (Tick)

	**Signs and symptoms**	**No**	**Slightly**	**Mildly**	**Moderate**	**Severe**
6.	Angry/Irritable/Frustrated	0	1	2	3	4
7.	Anxious/Nervous					
8.	Depressed mood/Sad					
9.	Difficulty concentrating					
10.	Increased appetite, hungry					
11.	Insomnia, sleep problems					
12.	Restless					
13.	Constipation					
14.	Coughing					
15.	Dizziness					

Monitoring:

1. Tobacco that you usually buy, is sold by a certified company
   A. No (0)
   B. Yes (1)

2. In your area, tobacco can be bought by a minor age person (less than 18 years)
   A. No (0)
   B. Yes (1)

3. Is the tobacco that you usually buy; Taxed?
   A. No (0)
   B. Yes (1)

Protecting:

1. I see signs of "NO SMOKING" or "NO TOBACCO" quite often
   A. No (0)
   B. Yes (1)

Offering:

1. I have been offered free tobacco quit-service (smoking/smokeless) by other people
   A. No (0)
   B. Yes (1)

2. I have offered free tobacco quit-service (smoking/smokeless) to other people
   A. No (0)
   B. Yes (1)

Warning:

1. The packaging of tobacco that you usually buy, displays harmful warnings of tobacco use
   A. No (0)
   B. Yes (1)

DR. QUADRI - COPYWRITE

2. Television and movies that you usually watch, display about the warnings of tobacco use
   A. No (0)
   B. Yes (1)

Enforcing:

1. I feel that the place where I live have strict regulations for the use of tobacco
   A. No (0)
   B. Yes (1)

2. I have seen tobacco advertised on streets / on television / during movies
   A. No (0)
   B. Yes (1)

Raising:

1. The tax on tobacco has been raised recently (within 5 years from now)
   A. No (0)
   B. Yes (1)

DR. QUADRI - COPYWRITE
