# Supplementary material for: Poor implementation of tobacco control measures and lack of education influences the intention to quit tobacco: a structural equation modelling approach
Source: BMC Public Health. 2022 Jun 15;22:1199. doi: 10.1186/s12889-022-13565-3 (PMC9202196; doi:10.1186/s12889-022-13565-3)
Supplement: Supplementary file 2 — Additional file 2. Sumplementary tables and figures. [file 12889_2022_13565_MOESM2_ESM.docx]

**Table S1. Linear mixed modelling analysis for factors associated with the ITQ**

|  | *Linear mixed Modelling* | | | *Linear regression analysis* | | |
| --- | --- | --- | --- | --- | --- | --- |
| *Predictors* | *Estimates* | *95% CI* | *p* | *Estimates* | *95% CI* | *p* |
| Sex: Female vs. Male | -0.11 | -0.33 – 0.11 | 0.345 | -0.13 | -0.36 – 0.09 | 0.246 |
| Age (1 year increase) | -0.01 | -0.02 – -0.01 | **<0.001** | -0.01 | -0.02 – -0.01 | **<0.001** |
| Education | 0.12 | 0.08 – 0.16 | **<0.001** | 0.12 | 0.08 – 0.16 | **<0.001** |
| Location: Semi-Urban | 0.44 | 0.29 – 0.59 | **<0.001** | 0.55 | 0.41 – 0.70 | **<0.001** |
| Location: Urban | 0.77 | 0.64 – 0.90 | **<0.001** | 0.89 | 0.76 – 1.01 | **<0.001** |
| MP (1 point increase) | 0.13 | 0.09 – 0.16 | **<0.001** | 0.14 | 0.11 – 0.18 | **<0.001** |
| Cigarette: Yes vs. No | -0.13 | -0.25 – -0.01 | **0.028** | -0.16 | -0.27 – -0.04 | **0.009** |
| Bidi: Yes vs. No | -0.29 | -0.41 – -0.17 | **<0.001** | -0.28 | -0.40 – -0.16 | **<0.001** |
| Shisha: Yes vs. No | -0.30 | -0.66 – 0.07 | 0.113 | -0.33 | -0.71 – 0.04 | 0.079 |
| Tambakoo: Yes vs. No | 0.34 | 0.23 – 0.46 | **<0.001** | 0.40 | 0.29 – 0.52 | **<0.001** |
| Betel: Yes vs. No | -0.11 | -0.28 – 0.06 | 0.198 | -0.12 | -0.29 – 0.06 | 0.190 |
| Influencers (N) | 0.17 | 0.11 – 0.22 | **<0.001** | 0.19 | 0.14 – 0.25 | **<0.001** |
| Duration (One level increase) | 0.03 | -0.03 – 0.09 | 0.316 | 0.04 | -0.02 – 0.10 | 0.224 |
| Frequency (One level increase) | -0.15 | -0.22 – -0.08 | **<0.001** | -0.17 | -0.25 – -0.10 | **<0.001** |
| Dependency (One level increase) | -0.13 | -0.24 – -0.02 | **0.020** | -0.10 | -0.22 – 0.01 | 0.071 |
| Linear mixed modelling was performed with the state as a random intercept  Estimates (B) represent the average change in intention to quit  CI: confidence interval | | | | | | |

**Figure S1. Regression analysis model diagnostics**

**Figure S2. Association between state and ITQ**

Only one respondent was from Mumbai and was excluded

**Figure S3. Association between state and MPower**

Only one respondent was from Mumbai and was excluded

**Table S2. Post-hoc pairwise comparisons for the association between state and MPower**

|  | Bihar | Kerela | Madhya prades | Maharashtra | Punjab | Telangana | Uttar pradesh |
| --- | --- | --- | --- | --- | --- | --- | --- |
| Bihar | 6.97 | 0.99 | **<.0001** | **0.0004** | 0.1352 | <.0001 | 1 |
| Kerala | -0.05 | 7.07 | **<.0001** | **<.0001** | **0.0267** | <.0001 | 1 |
| Madhya pradesh | 0.98 | 1.07621 | 5.99 | **0.0011** | **<.0001** | 1 | 0.46 |
| Maharashtra | 0.49 | 0.59 | -0.49 | 6.48 | 0.53 | 0.0859 | 0.95 |
| Punjab | 0.31 | 0.40 | -0.67 | -0.18 | 6.67 | **0.0038** | 0.99 |
| Telangana | 0.97 | 1.07 | -0.01 | 0.48 | 0.67 | 6 | 0.50 |
| Uttar pradesh | -0.03 | 0.068 | -1.01 | -0.52 | -0.33 | -1 | 7 |
| Diagonals represent the averge MPower score  P values were corrected using the Tukey adjustment  The upper riangle represents the p values for post-hoc pair wise comparisons between the means scores of the row and column headers  The lower triangle represent the difference in the means between the row and column headers | | | | | | | |

**Table S3. Association between influencer and intention to quit**

|  | **No one** | **Parents** | **Relatives** | **Friends** | **HCP** | **P** |
| --- | --- | --- | --- | --- | --- | --- |
|  | ***N=563*** | ***N=304*** | ***N=358*** | ***N=99*** | ***N=348*** |  |
| ITQ |  |  |  |  |  | <0.001 |
| Now | 205 (35.3%) | 92 (15.8%) | 110 (18.9%) | 39 (6.71%) | 135 (23.2%) |  |
| In the next 6 months | 117 (33.6%) | 80 (23.0%) | 75 (21.6%) | 24 (6.90%) | 52 (14.9%) |  |
| Sometime in the future beyond 6 months | 19 (11.4%) | 44 (26.3%) | 67 (40.1%) | 8 (4.79%) | 29 (17.4%) |  |
| Not decided | 188 (40.8%) | 88 (19.1%) | 95 (20.6%) | 24 (5.21%) | 66 (14.3%) |  |
| Never | 34 (29.6%) | 0 (0.00%) | 11 (9.57%) | 4 (3.48%) | 66 (57.4%) |  |
| ITQ: Intention to quit  HCP: Healthcare provider | | | | | | |

**Table S4. Average symptom score across each category of ITQ**

|  | Now | In the next 6 months | Sometime in the future beyond 6 months | Not decided | Never | p.overall |
| --- | --- | --- | --- | --- | --- | --- |
|  | *N=1007* | *N=424* | *N=186* | *N=543* | *N=127* |  |
| Angry/Irritable/Frustrated | 1.65 (1.01) | 1.51 (0.83) | 1.77 (1.03) | 1.78 (0.91) | 1.88 (1.21) | <0.001 |
| Anxious/Nervous | 1.71 (1.01) | 1.51 (0.86) | 1.54 (0.86) | 1.62 (0.88) | 1.31 (0.64) | <0.001 |
| Depressed mood/Sad | 1.61 (0.96) | 1.43 (0.77) | 1.49 (0.83) | 1.47 (0.76) | 1.06 (0.24) | <0.001 |
| Difficulty concentrating | 1.64 (0.99) | 1.43 (0.79) | 1.43 (0.73) | 1.62 (0.86) | 1.61 (1.09) | <0.001 |
| Increased appetite, hungry | 1.30 (0.75) | 1.32 (0.72) | 1.33 (0.82) | 1.30 (0.61) | 1.03 (0.18) | 0.001 |
| Insomnia, sleep problems | 1.33 (0.75) | 1.33 (0.76) | 1.32 (0.72) | 1.45 (0.82) | 1.12 (0.32) | <0.001 |
| Restless | 1.23 (0.60) | 1.21 (0.56) | 1.26 (0.63) | 1.42 (0.74) | 1.03 (0.18) | <0.001 |
| Constipation | 1.16 (0.53) | 1.24 (0.57) | 1.24 (0.54) | 1.38 (0.66) | 1.06 (0.23) | <0.001 |
| Coughing | 1.23 (0.60) | 1.12 (0.42) | 1.17 (0.46) | 1.32 (0.65) | 1.12 (0.41) | <0.001 |
| Dizziness | 1.12 (0.42) | 1.12 (0.42) | 1.27 (0.65) | 1.24 (0.57) | 1.03 (0.18) | <0.001 |
| Statistical analysis was performed using one-way ANOVA  Data was summarized using mean ± SD | | | | | | |

**Table S5. Effects of predictor variables on intention to quit tobacco using education as a multinomial variable**

| DV | IV | Std B | Z | P |
| --- | --- | --- | --- | --- |
| Dependency | MP | 0.033 | 1.417 | 0.156 |
|  | Bidi | 0.075 | 3.275 | 0.001 |
|  | Shisha | 0.110 | 4.950 | < 0.001 |
|  | Betel | 0.062 | 2.808 | 0.005 |
|  | No education | Ref |  |  |
|  | Primary education | 0.073 | 2.521 | 0.012 |
|  | Intermediate education | 0.068 | 2.347 | 0.019 |
|  | High school | 0.092 | 3.472 | 0.001 |
|  | Graduate | -0.074 | -2.845 | 0.004 |
|  | Post-graduate | -0.018 | -0.780 | 0.436 |
| Duration | MP | -0.069 | -3.160 | 0.002 |
|  | Bidi | 0.201 | 9.266 | < 0.001 |
|  | No education | Ref |  |  |
|  | Primary education | -0.150 | -5.477 | < 0.001 |
|  | Intermediate education | -0.214 | -7.809 | < 0.001 |
|  | High school | -0.233 | -9.207 | < 0.001 |
|  | Graduate | -0.179 | -7.222 | < 0.001 |
|  | Post-graduate | -0.084 | -3.922 | < 0.001 |
| Frequency | MP | -0.023 | -1.030 | 0.303 |
|  | Tambakoo | -0.128 | -5.687 | < 0.001 |
|  | Bidi | 0.149 | 6.346 | < 0.001 |
|  | Cigarette | -0.056 | -2.543 | 0.011 |
|  | No education | Ref |  |  |
|  | Primary education | -0.042 | -1.492 | 0.136 |
|  | Intermediate education | -0.117 | -4.123 | < 0.001 |
|  | High school | -0.106 | -4.059 | < 0.001 |
|  | Graduate | 0.035 | 1.352 | 0.176 |
|  | Post-graduate | -0.058 | -2.638 | 0.008 |
| ITQ | MP | 0.161 | 7.589 | < 0.001 |
|  | Frequency | -0.134 | -6.331 | < 0.001 |
|  | Dependency | 0.006 | 0.285 | 0.776 |
|  | Duration | -0.019 | -0.875 | 0.382 |
|  | Influencers | -0.652 | -1.519 | 0.129 |
|  | Cigarette | -0.054 | -2.604 | 0.009 |
|  | Bidi | -0.118 | -5.421 | < 0.001 |
|  | Tambakoo | 0.186 | 8.609 | < 0.001 |
|  | Rel | 0.382 | 1.800 | 0.072 |
|  | Parents | 0.271 | 1.463 | 0.143 |
|  | HCP | 0.408 | 1.993 | 0.046 |
|  | Friends | 0.406 | 2.331 | 0.020 |
|  | No education | Ref |  |  |
|  | Primary education | 0.082 | 3.191 | 0.001 |
|  | Intermediate education | 0.088 | 3.330 | 0.001 |
|  | High school | 0.143 | 5.870 | < 0.001 |
|  | Graduate | 0.097 | 4.067 | < 0.001 |
|  | Post-graduate | 0.093 | 4.661 | < 0.001 |
